# Intracellular interplay between cholecystokinin and leptin signalling for satiety control in rats

**DOI:** 10.1038/s41598-020-69035-6

**Published:** 2020-07-20

**Authors:** Hayato Koizumi, Shahid Mohammad, Tomoya Ozaki, Kiyokazu Muto, Nanami Matsuba, Juhyon Kim, Weihong Pan, Eri Morioka, Takatoshi Mochizuki, Masayuki Ikeda

**Affiliations:** 1 Graduate School of Innovative Life Science, University of Toyama, 3190 Gofuku, Toyama, 930-8555 Japan; 2 Graduate School of Science and Engineering, University of Toyama, 3190 Gofuku, Toyama, 930-8555 Japan; 3 Center for Neuroscience Research, Children’s National Medical Center, Washington, DC 20010 USA; 4 Graduate School of Medicine, Nagoya University, 65 Tsurumaicho, Showa-ku, Nagoya, 466-8550 Japan; 5 Blood-Brain Barrier Group, Pennington Biomedical Research Center, 6400 Perkins Road, Baton Rouge, LA 70808 USA; 6 Present Address: Biopotentials Consulting, Sedona, AZ 86351 USA

**Keywords:** Hypothalamus, Obesity

## Abstract

Cholecystokinin (CCK) and leptin are satiety-controlling peptides, yet their interactive roles remain unclear. Here, we addressed this issue using in vitro and in vivo models. In rat C6 glioma cells, leptin pre-treatment enhanced Ca^2+^ mobilization by a CCK agonist (CCK-8s). This leptin action was reduced by Janus kinase inhibitor (AG490) or PI3-kinase inhibitor (LY294002). Meanwhile, leptin stimulation alone failed to mobilize Ca^2+^ even in cells overexpressing leptin receptors (C6-ObRb). Leptin increased nuclear immunoreactivity against phosphorylated STAT3 (pSTAT3) whereas CCK-8s reduced leptin-induced nuclear pSTAT3 accumulation in these cells. In the rat ventromedial hypothalamus (VMH), leptin-induced action potential firing was enhanced, whereas nuclear pSTAT3 was reduced by co-stimulation with CCK-8s. To further analyse in vivo signalling interplay, a CCK-1 antagonist (lorglumide) was intraperitoneally injected in rats following 1-h restricted feeding. Food access was increased 3-h after lorglumide injection. At this timepoint, nuclear pSTAT3 was increased whereas c-Fos was decreased in the VMH. Taken together, these results suggest that leptin and CCK receptors may both contribute to short-term satiety, and leptin could positively modulate CCK signalling. Notably, nuclear pSTAT3 levels in this experimental paradigm were negatively correlated with satiety levels, contrary to the generally described transcriptional regulation for long-term satiety via leptin receptors.

## Introduction

Food intake control is essential for animal survival, and multiple signalling mechanisms are involved in intake behavior^1,2^. Cholecystokinin (CCK), which is a peptide hormone secreted from the intestine in response to food intake^3–5^, is the classic satiety-controlling molecules^6,7^. The dominant receptor subtypes, CCK-1 and CCK-2 receptors, are both G_q_-coupled seven-transmembrane receptors^8,9^. CCK-1 receptors influence satiety, as CCK-1 agonists reduce food intake^10,11^ whereas pharmacological blockage of CCK-1 receptors stimulate food intake^10,12–19^. CCK-1 receptors are localized in peripheral and central machinery known to control satiety, and their dense expression has been found in the pylorus, nodose ganglion, nucleus tractus solitarius, and hypothalamic satiety-controlling centres^20^. Both peripheral and central administration of CCK induce satiety responses^21,22^, although permeability of peripheral CCK to the brain is still a matter of controversy. Hypothalamic neurons contain high levels of CCK peptides^23,24^ and the central role of CCK to suppress food intake is reported to be dependent on neural circuits expressing CCK-1 receptors^25–29^. The function of hypothalamic CCK-1 receptors is supported by CCK-2 receptors, as demonstrated by their functional compensation evident in CCK-1 receptor knockout mice^30^. Whether such direct receptor-wide interactions could be present among other receptors in the brain is currently unknown. In the present study, we investigated the intracellular interaction between CCK signaling and another peptide that mediates food intake, leptin.

A product of the obese gene, leptin, is a peptide hormone secreted from white adipocyte tissue, also known as a satiety-controller^31–33^. Unlike CCK, leptin reduces meal size but does not alter meal frequency^34,35^, suggesting a more long-term effect on body weight regulation via leptin signalling. Splice variants (ObRa–ObRe) have been identified for leptin receptors and only the full-length isoform (ObRb) contains the intracellular motifs required for the initiation of intracellular signalling^36^. ObRb is a single-transmembrane receptor that interacts with diverse signalling molecules^37–40^ including Janus kinase-2 (JAK2), signal transducer and activator of transcription-3 (STAT3), phosphoinositide-3 kinase (PI3K), mitogen-activated protein kinase (MAPK), and 5′-AMP-activated protein kinase (AMPK). It has also been shown that leptin modulates cytosolic Ca^2+^ signalling, although the action seems to be variable among cell types. For example, leptin exerts excitatory actions on *N*-methyl-d-aspartate-induced Ca^2+^ influx via MAPK pathways in cerebellar granule cell cultures^41^. Leptin receptors are expressed in the hypothalamic ventromedial nucleus (VMN) to control satiety^42^. Indeed, leptin increased or decreased intrinsic Ca^2+^ oscillations depending on cell populations in primary cultures of ventromedial hypothalamus (VMH), which include the VMN and arcuate nucleus (ARC) neurons^40^. The inhibitory action of leptin is mediated by K^+^ channel activation via AMPK whereas the excitatory action of leptin is yet to be characterized^40^. In addition, ghrelin-induced Ca^2+^ elevation in dissociated ARC neurons, which depend on the gating of N-type Ca^2+^ channels, were inhibited by leptin^43^. Furthermore, hypothalamic astrocyte cultures demonstrated monophasic cytosolic Ca^2+^ elevation following leptin stimulation^44^ although the mechanisms were not characterized. Glial leptin function may also be involved in satiety control because conditional knockout of leptin receptors in astrocytes diminished the action of leptin to suppress food intake^45^.

Intriguingly, co-administration of sub-threshold CCK and leptin, which individually have no effect on feeding, dramatically reduced food intake in mice^46^ and rats^47,48^. Also, the effect of CCK on satiety control is attenuated in Zucker fa/fa rats lacking functional leptin receptors^49^. In addition, other evidence implicated that leptin-mediated satiety-controlling systems may strongly interact with CCK systems^18,50–54^. For example, c-Fos expression in oxytocin neurons in the hypothalamic paraventricular nucleus is elevated by leptin administration, and these neurons have axons that extend to the nucleus tractus solitarius where peripheral CCK signals are received^52^. However, a fourth ventricular injection of leptin enhanced the CCK-induced satiety response but failed to increase c-Fos expression in the nucleus tractus solitarius^18^. Thus, CCK and leptin could synergistically regulate satiety via a large neural network. Furthermore, the nodose ganglion neurons co-express ObRb and CCK-1 receptors, and their synergistic excitatory actions were identified in these neurons^53,54^. This suggests a direct interaction of these receptors within peripheral satiety controllers, although it is still unclear as to how these receptors interact at the intracellular signalling level in the central nervous system. Based on these findings, the present study attempted to address this question using various models.

## Results

### Rat C6 glioma as a model cell line

**Figure 1:**
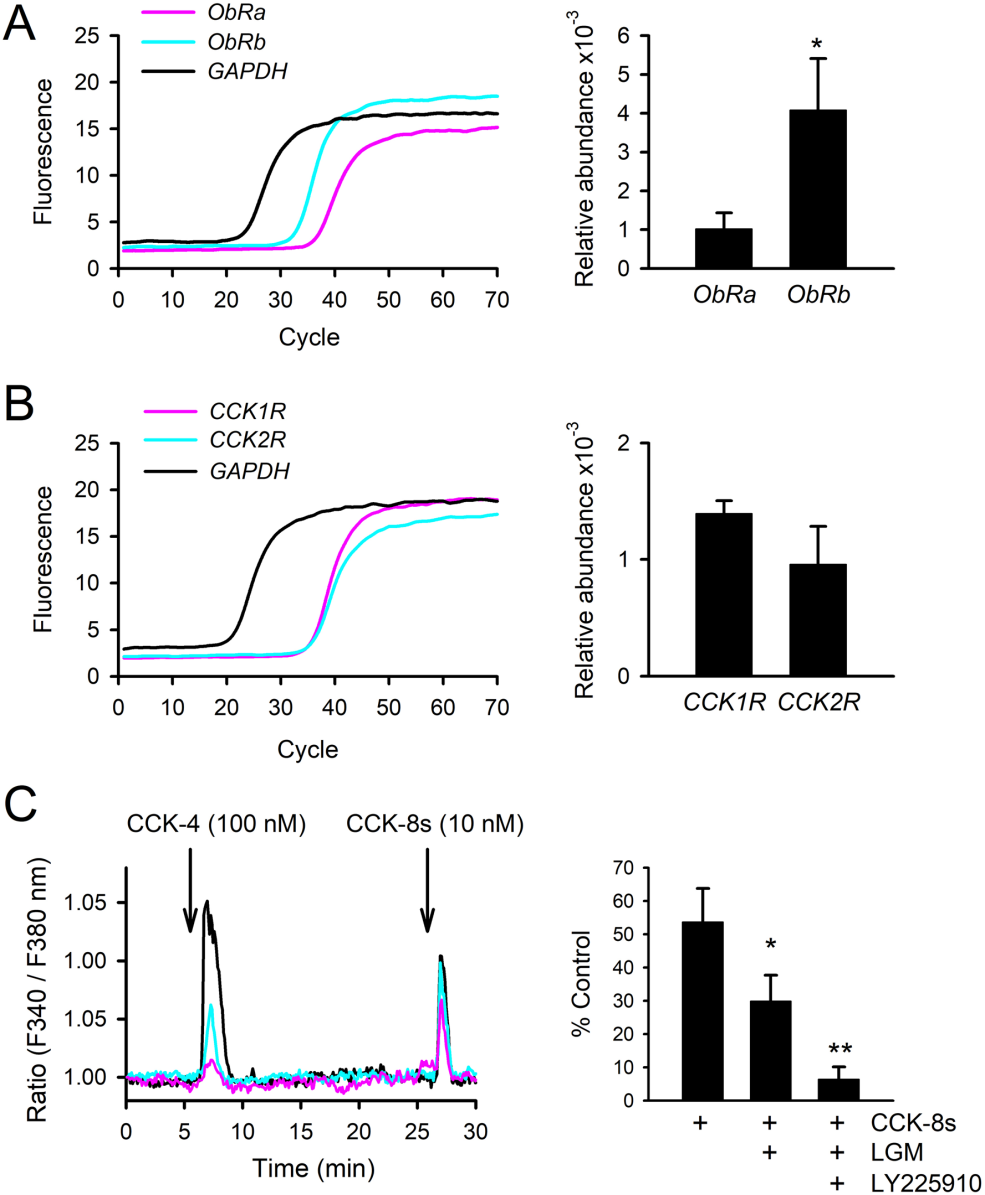
Leptin and CCK receptors are intrinsically expressed in rat C6 glioma cells. (**A**) Real time RT-PCR for *ObRa*, *ObRb*, and a housekeeping gene (glyceraldehyde-phosphate dehydrogenase, *GAPDH*) are shown. Accordingly, relative abundance of mRNA was quantified. Relatively higher expression of *ObRb* was observed. *p < 0.05 by Student’s *t*-test. (**B**) RT-PCR analysis of CCK-1 receptor (*CCK1R*) and CCK-2 receptor (*CCK2R*) mRNA. (**C**) Cytosolic Ca^2+^ concentrations were analyzed by the Fura-2 ratio-metric technique. Cytosolic Ca^2+^ was elevated by CCK-4 (100 nM) and CCK-8s (10 nM) in C6 cells. Arrows denote the onset of 45-s pulses of these drug applications. Three representative cell responses are shown as black, pink, and blue traces. The CCK-8s-induced Ca^2+^ response was analyzed under the perfusion of CCK-1 receptor antagonists (LGM, 100 nM) and LGM plus CCK-2 antagonist (LY225910, 100 nM). *p < 0.05 and **p < 0.01 compared with the magnitude of Ca^2+^ response without antagonist perfusion by one-way ANOVA followed by Duncan’s multiple range test.

As in hypothalamic astrocytes, C6 cells have been reported to express ObRa (short-length isoform) and ObRb (full-length isoform), with a higher proportion of ObRa^44,55^. As C6 cell is a cancer cell line, we first tested expression in our laboratory C6 cell stocks. Using real time RT-PCR, the present study confirmed the expression of *ObRa* and *ObRb*, with *ObRb* expression levels being four times higher (*p* < 0.05 by Student’s *t*-test; Fig. 1A), suggesting the ratio may be variable among stored samples or between cell passages.

Since CCK ligand binding has also been reported in C6 cells^56^, the present study analyzed the gene expression of CCK-1 and CCK-2 receptors using real time RT-PCR. Both genes seemed to be expressed in C6 cells (Fig. 1B). To further confirm CCK receptor function, C6 cells were analyzed by Ca^2+^ imaging. Stimulation of C6 cells with CCK-2 receptor agonist, CCK-4 (100 nM), evoked Ca^2+^ transients in approximately 20% of C6 cells (n = 35/three dishes) whereas the size of the Ca^2+^ response largely varied among cells. When the same cells were re-stimulated with less-specific agonist, CCK-8s (10 nM), 32% of C6 cells displayed Ca^2+^ transients (n = 103/five dishes). Pretreatment with CCK-1 receptor antagonist, lorglumide (LGM, 100 nM) significantly reduced the magnitude of CCK-8s-induced Ca^2+^ transients (− 44%, F_2,24_, *p* < 0.05 by one-way ANOVA; Fig. 1C). Additional pretreatment with CCK-2 receptor antagonist, LY225910 (100 nM), almost completely reduced the CCK-8s-induced Ca^2+^ transients (F_2,24_, *p* < 0.01 by one-way ANOVA; Fig. 1C). These results indicated intrinsic expression of CCK-1 and CCK-2 receptors in a variety of subtype ratios in C6 cells.

### Signalling interactions for Ca^2+^ mobilization in C6 cells

**Figure 2:**
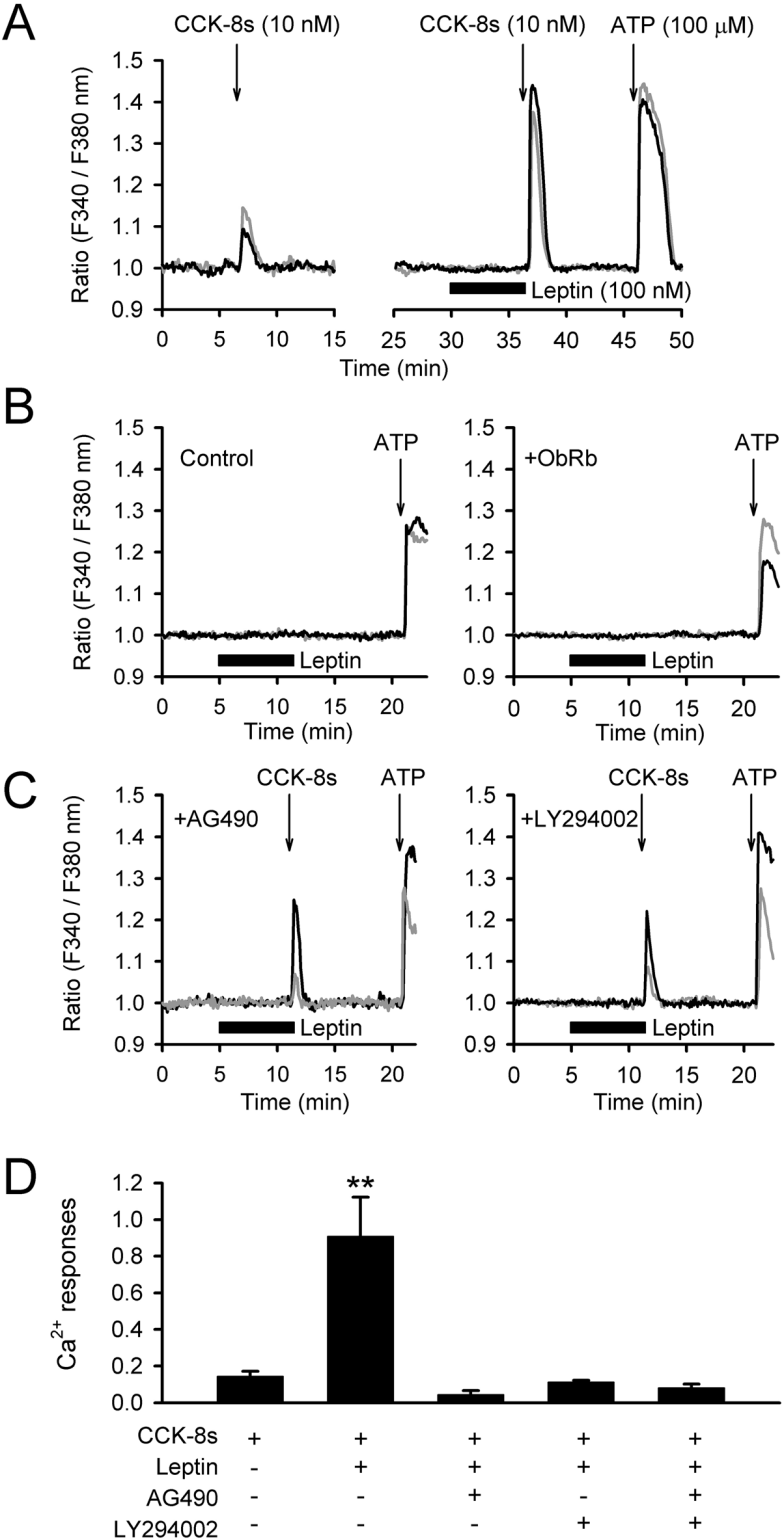
Cytosolic Ca^2+^ mobilization in C6 cells. (**A**) The CCK-8s (10 nM)-induced Ca^2+^ transients in C6 cells were amplified by pre-treatment with leptin (100 nM). High Ca^2+^ mobilization via ATP (100 µM) at the end of experiment was observed as positive control. Two representative cell responses are shown as black and grey traces. (**B**) Leptin stimulation alone failed to evoke cytosolic Ca^2+^ responses in wild-type C6 cells (Control, left) and C6 cells overexpressing mouse leptin receptors (+ ObRb, right). (**C**) C6 cells were pre-incubated with JAK inhibitor (1 μM AG490, left) or PI3K inhibitor (50 µM LY294002, right) for 10 min before starting Ca^2+^ imaging. These kinase inhibitors significantly reduced leptin action for CCK-8s-induced Ca^2+^ mobilization. (**D**) The magnitude of Ca^2+^ responses was quantified and compared. **p < 0.01 by one-way ANOVA.

Ca^2+^ imaging studies were further used to test the effects of leptin on CCK-8s (10 nM)-induced Ca^2+^ mobilization. The results indicated significant (~ 5 times) amplification in the magnitude of Ca^2+^ mobilization by leptin (100 nM, 5 min) pre-treatment (n = 115/five dishes; Fig. 2A,D). Meanwhile, leptin (100 nM, 5 min as in Fig. 2B or 500 nM, 5 min) treatment failed to evoke Ca^2+^ transients in wild-type C6 cells (n = 185/eight dishes) and C6 cells stably transfected with mouse *ObRb* (C6-ObRb; n = 190/11 dishes). In addition, the magnitude of Ca^2+^ response by co-stimulation of leptin (100 nM) and CCK-8s (10 nM) in C6-ObRb (n = 84/four dishes) was not significantly different from that in wild-type C6 cells (n.s. by Student’s *t*-test). When wild-type C6 cells were incubated for 10 min with a JAK inhibitor, AG490 (1 μM), leptin failed to amplify the CCK-8s-induced Ca^2+^ mobilization (n = 48/three dishes; Fig. 2C,D). Also, the same treatment with a PI3K inhibitor, LY294002 (50 µM), significantly inhibited CCK-8s-induced Ca^2+^ mobilization (n = 45/three dishes; Fig. 2C,D). These results indicate amplification of CCK signalling by leptin signalling in C6 cells.

### Signalling interaction for STAT3 phosphorylation in C6 cells

**Figure 3:**
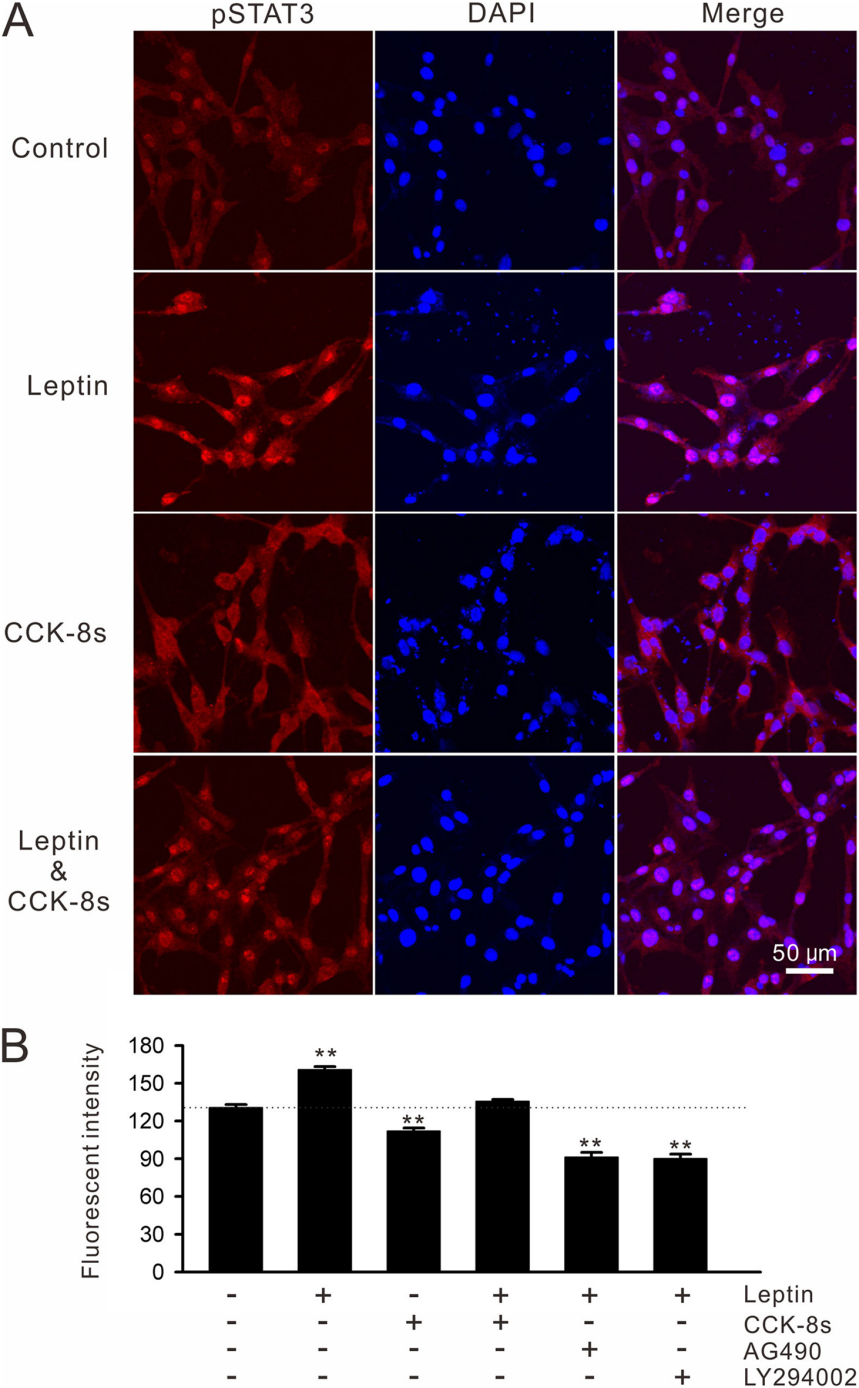
Immunostaining of pSTAT3 in C6-ObRb cells. (**A**) C6-ObRb cells were stimulated for 1–2 min with leptin (100 nM) or CCK-8s (10 nM) 30 min prior to fixation. pSTAT3 (red) images were merged with DAPI nuclear staining (blue) on the right. Note that leptin stimulation increased nuclear pSTAT3 whereas CCK-8s rather increased cytosolic pSTAT3. Co-stimulation with leptin and CCK-8s resulted in nuclear pSTAT3 expression whereas the level was not different from un-stimulated controls. (**B**) Nuclear fluorescent intensities of pSTAT3 were compared. AG490 (1 µM) or LY294002 (50 µM) blocked leptin-induced nuclear pSTAT3 elevation. **p < 0.01 by Duncan’s multiple range test following one-way ANOVA.

Immunofluorescent staining was examined to visualize pSTAT3 in C6 cells following receptor stimulation. In this study, C6-ObRb was used as a model cell line to maximize pSTAT3 immunoreactivity (ir). There was detectable nuclear pSTAT3-ir in un-stimulated C6-ObRb cells (Fig. 3A). Stimulation with leptin (100 nM) significantly increased pSTAT3-ir in the nuclei of C6-ObRb (30 images/three dishes, F_5,174_ = 119.4, *p* < 0.01 by one-way ANOVA; Fig. 3A,B). Interestingly, stimulation with CCK-8s (50 nM) induced cytosolic pSTAT3-ir in C6-ObRb (Fig. 3A). The nuclear intensity of pSTAT3-ir, in turn, was significantly smaller in the CCK-8s-treated group than in unstimulated controls (30 images/three dishes, F_5,174_ = 119.4, *p* < 0.01 by one-way ANOVA; Fig. 3A,B), suggesting cytosolic immobilization of pSTAT3 signals. Intriguingly, co-stimulation with leptin (100 nM) and CCK-8s (50 nM) induced nuclear pSTAT3-ir whereas the intensity was significantly smaller than that by leptin stimulation alone (30 images/three dishes, F_5,174_ = 119.4, *p* < 0.01 by one-way ANOVA; Fig. 3A,B). The effect of AG490 (1 µM) or LY294002 (50 µM) was also analyzed as for the Ca^2+^ imaging assays, and these kinase inhibitors significantly reduced nuclear pSTAT3-ir below the level of un-stimulated controls (30 images/three dishes, F_5,174_ = 119.4, *p* < 0.01 by one-way ANOVA; Fig. 3B). Unlike CCK-8s stimulation, cytosolic pSTAT3 signal was not elevated by these kinase inhibitors.

### Activation of VMN neurons by co-stimulation of CCK and leptin receptors

**Figure 4:**
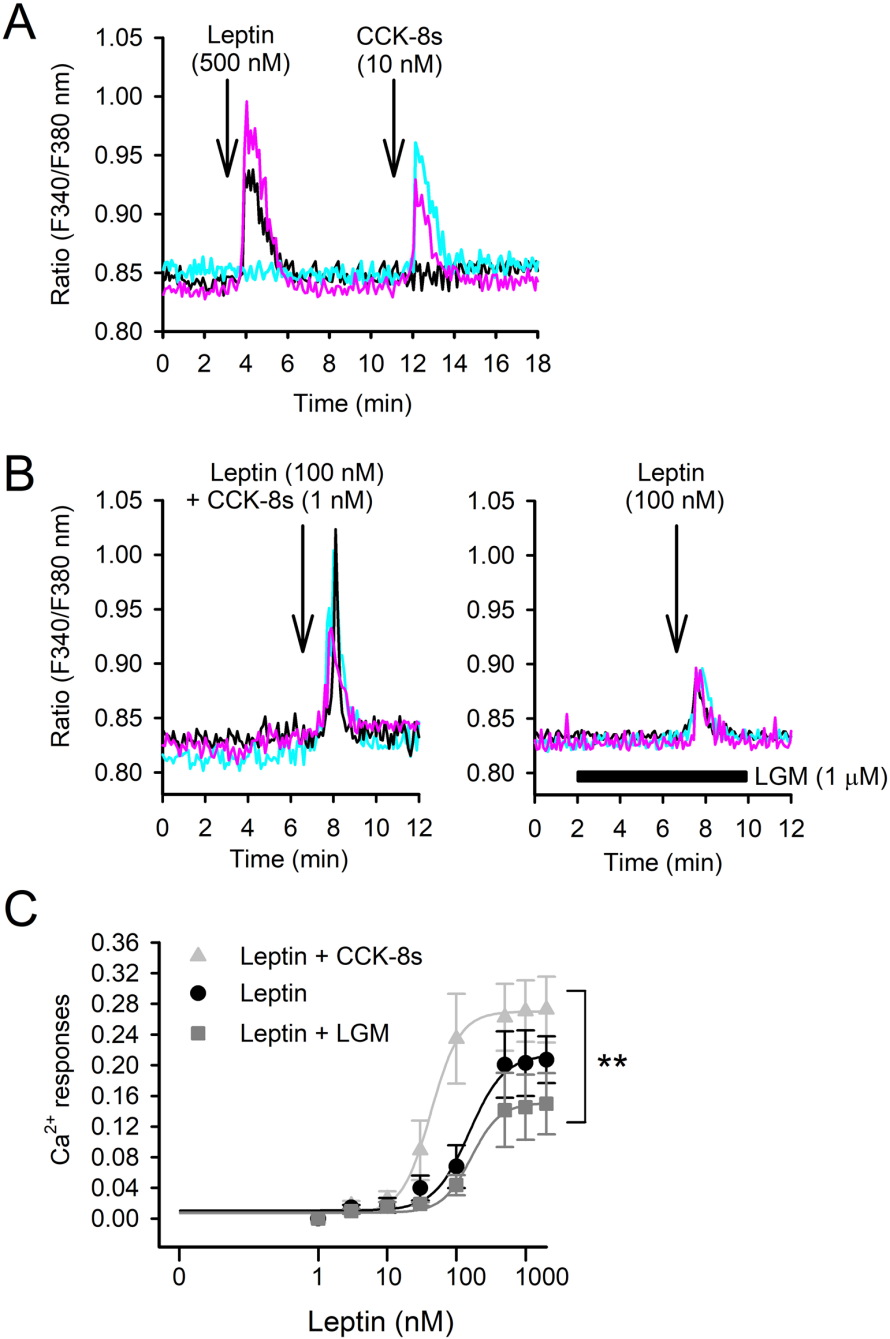
Cytosolic Ca^2+^ mobilization in the VMN. (**A**) VMN cells were repeatedly stimulated by 500 nM leptin and 10 nM CCK-8s. Three representative cell responses are shown. Note that there are three populations that displayed increased Ca^2+^ solely by leptin (black) or CCK-8s (blue) or by both (pink). (**B**) Left: representative cell responses following co-stimulation with 100 nM leptin and 1 nM CCK-8s. Right: Representative cell responses following stimulation with 100 nM leptin under the treatment of 1 µM LGM. (**C**) Concentration response curves for leptin (R_max_ = 0.2, EC_50_ = 152.9 nM). The response curve was shifted up by co-stimulation of 1 nM CCK-8s (R_max_ = 0.26, EC_50_ = 42.8 nM) and shifted down by blocking CCK-1 receptors by 1 µM LGM (R_max_ = 0.14, EC_50_ = 162.7 nM). **p < 0.01 by two-way ANOVA.

To further analyze the synergistic functions of leptin and CCK receptors for satiety-controlling neurons, rat VMH slices were analyzed by the Ca^2+^ imaging technique. Unlike the case of C6 cells, high leptin (500 nM) concentration evoked a monophasic Ca^2+^ increase in cells in the VMN (65.4 ± 7.5%, 11 slices; Fig. 4A) and the ARC (25.9% ± 4.5%, 12 slices). In the VMN, the majority of the leptin responders also responded to 10 nM CCK-8s to increase intracellular Ca^2+^ (81.3 ± 9.1%, 11 slices), although there were also cells that exclusively responded to leptin (12.2 ± 1.5%, 11 slices) or CCK-8s (5.2 ± 1.1%, 11 slices; Fig. 4A). The present study examined stimulation of VMN cells with leptin (1–1,000 nM) together with 1 nM CCK-8s or after treatment with 1 µM LGM (Fig. 4B,C). Although 1 nM CCK-8s failed to evoke Ca^2+^ transients in slice preparation (n = 216 in 18 slices), co-stimulation amplified leptin-induced Ca^2+^ responses (F_2,16_ = 7.09, *p* < 0.01 by two-way ANOVA; Fig. 4C). On the contrary, LGM treatment reduced leptin-induced Ca^2+^ responses (F_2,16_ = 7.09, *p* < 0.01 by two-way ANOVA; Fig. 4C).

**Figure 5:**
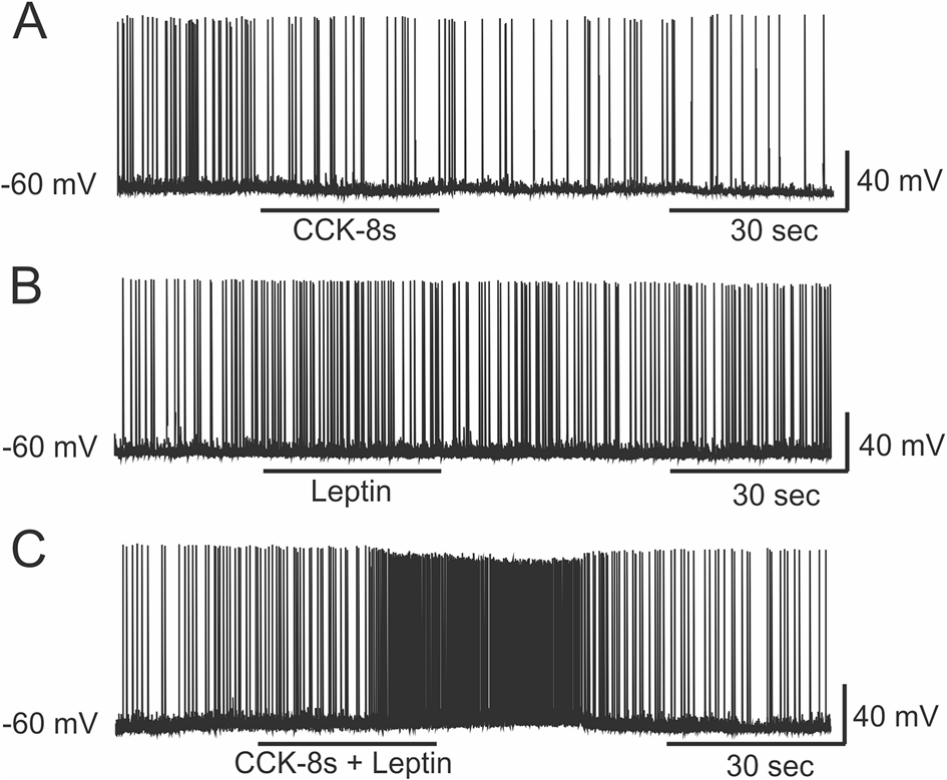
Action potential firing in VMN neurons. (**A**) Spontaneous action potential firing frequencies were not changed by the application of a sub-threshold concentration of CCK-8s (1 nM). (**B**) Also, leptin (30 nM) had little effect on firing frequencies. (**C**) Co-stimulation with CCK-8s (1 nM) and leptin (30 nM) significantly increased firings. (**B**, **C**) were recorded from a single neuron with a 15-min gap under continuous perfusion of ACSF.

Whole cell patch clamp study was also performed in rat VMN neurons to monitor action potential firing. When the holding potential was set at − 60 mV, the average spontaneous action potential firing frequency was 1.45 ± 0.07 Hz (n = 9). Application of 1 nM CCK-8s had little effect on action potential firing frequencies (1.28 ± 0.15 Hz, n = 9, F_3,32_ = 8.46, n.s. by one-way ANOVA followed by Duncan’s multiple range test; Fig. 5A). Furthermore, 30 nM leptin had little effect on action potential firing frequencies (1.55 ± 0.13 Hz, n = 9, F_3,32_ = 8.46, n.s. by one-way ANOVA followed by Duncan’s multiple range test; Fig. 5B). However, a significant increase in action potential firing frequencies was observed following co-stimulation with 1 nM CCK-8s and 30 nM leptin (3.49 ± 0.68 Hz, n = 9, F_3,32_ = 8.46, *p* < 0.01 by one-way ANOVA followed by Duncan’s multiple range test; Fig. 5C). Five neurons in nine recordings displayed apparent (> twofold) increases in frequencies, with the highest frequency reaching 5.5 Hz (Fig. 5C).

### Signalling interplay in vivo

**Figure 6:**
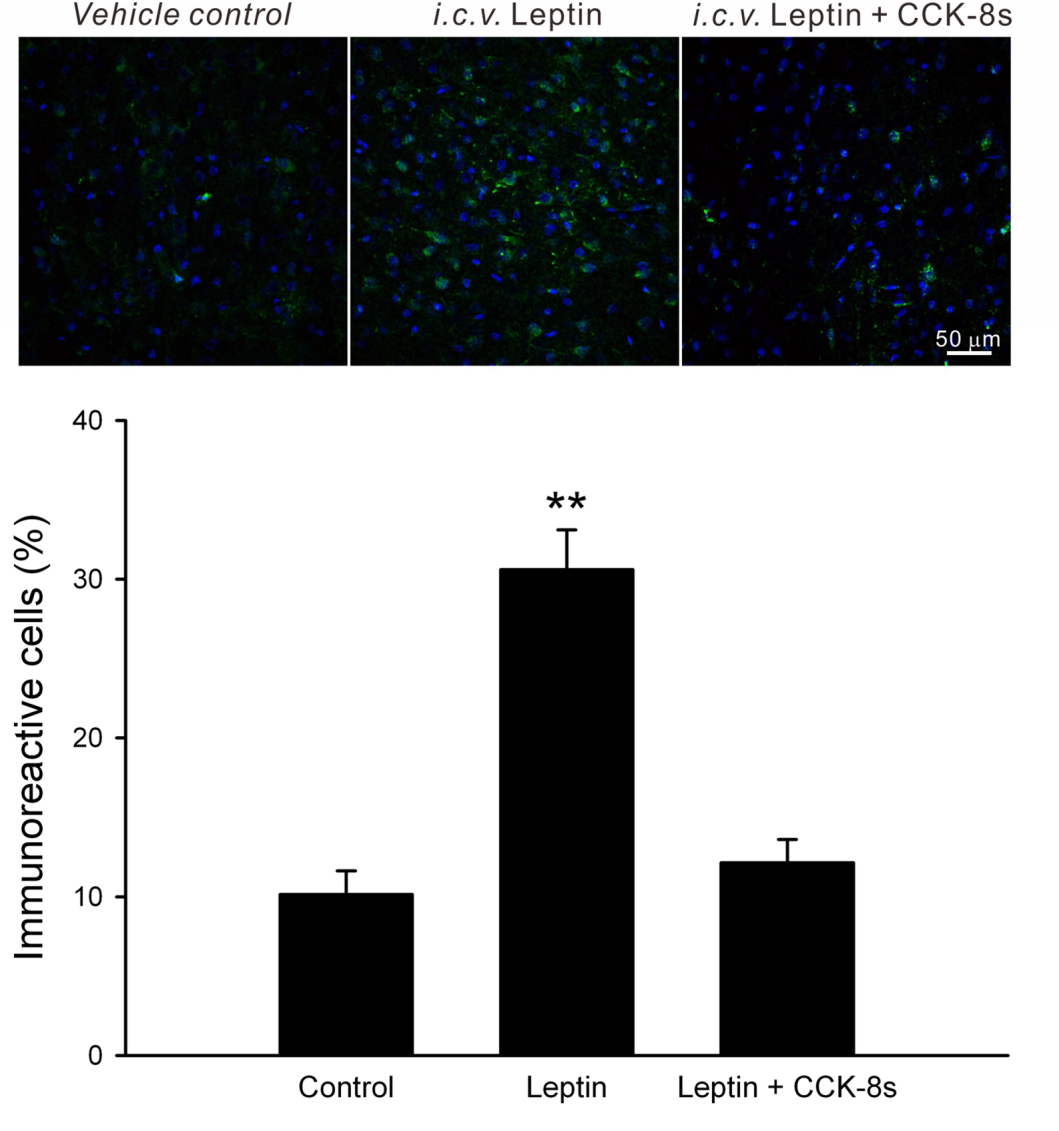
Effects of *i.c.v*. leptin and CCK on pSTAT3-ir in the rat VMN. Leptin (4 µg) or leptin plus CCK-8s (2 µg) was injected into the rat lateral ventricle 1 h prior to brain removal and fixation. pSTAT3 (green) images were merged with DAPI nuclear staining (blue). Compared with the vehicle (4 µl ACSF)-injected controls, nuclear pSTAT3-ir in the VMN was enhanced by leptin injections. Note that co-administration of leptin and CCK-8s failed to increase nuclear pSTAT3-ir in the VMN. Average number of nuclear pSTAT3-ir was analysed; 12 image frames were used to calculate the average. **p < 0.01 by Duncan’s multiple range test following one-way ANOVA.

To investigate the signalling interplay in vivo, the following two sets of experiments were performed. First, to observe direct interactions, nuclear pSTAT3-ir in the rat VMN were visualised by immunohistochemistry following intracerebroventricular (*i.c.v.*) injection of leptin (4 µg) or leptin with CCK-8s (2 µg). To minimize the effect of endogenous signals, all rats were deprived of food for 10 h before the *i.c.v.* administration. Under these circumstances, *i.c.v.* injection of leptin significantly increased number of nuclear pSTAT3-ir (three times that of vehicle-injected control, F_2,33_ = 36.03, *p* < 0.01 by one-way ANOVA followed by Duncan’s multiple range test; Fig. 6). Notably, leptin injection together with CCK-8s failed to increase the number of nuclear pSTAT3-ir in the VMN (n.s. by one-way ANOVA followed by Duncan’s multiple range test; Fig. 6), consistent with the results using C6 models (Fig. 3B).

**Figure 7:**
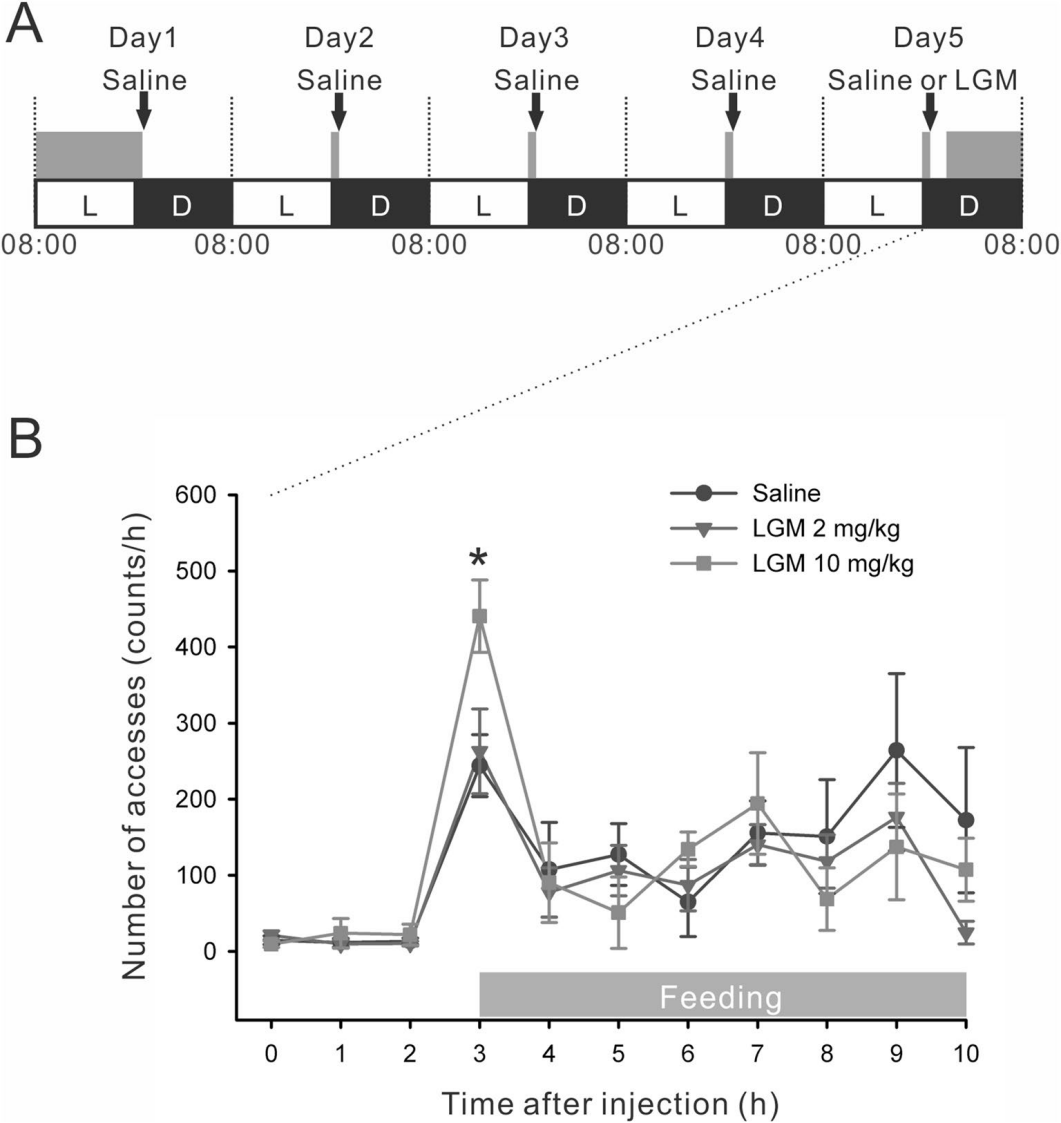
Food access frequency following *i.p.* injection of LGM in rats. (**A**) Experimental schedule for RF and *i.p.* injections for the estimation of satiety responses. Rats were acclimatized daily for 1-h RF and *i.p.* injections of saline. Grey bar denotes the timing of feeding. *L* light period, *D* dark period. (**B**) On day 5, rats were injected with saline or LGM (2 or 10 mg/kg) and then allowed to re-feed with a 3-h gap. Hourly number of accesses to the food chamber was counted. n = 5–6 for each group, *p < 0.05 by repeated one-way ANOVA.

Second, to address the signalling interplay under more physiological realistic conditions, systemic blockage of CCK-1 receptors was examined in rats. However, intraperitoneal (*i.p.*) injection of CCK-1 receptor antagonist stimulates food intake^10,12–19^ and thus *i.p.* injection of LGM will influence intrinsic CCK and leptin release due to the size of meal or energy intake. To stabilise conditions, rats were acclimatized to 1-h restricted feeding (RF) for 5 days as shown in Fig. 7A. During the 5-day RF period, the average body weight of rats was moderately reduced (− 8.1 ± 0.9%) whereas the amount of daily food intake was stabilised from day 4 (Fig. S1). Rats were also adapted to a daily injection of saline after the RF. On day 5, saline or LGM (2 mg/kg or 10 mg/kg) were injected. Three hours after the final injection, rats were allowed re-feeding to count the frequencies of food access. Under these circumstances, food access frequency at the onset of re-feeding was significantly increased by 10 mg/kg LGM injection (1.8 times that of the saline control, F_2,14_ = 4.63, *p* < 0.05 by repeated one-way ANOVA; Fig. 7B).

**Figure 8:**
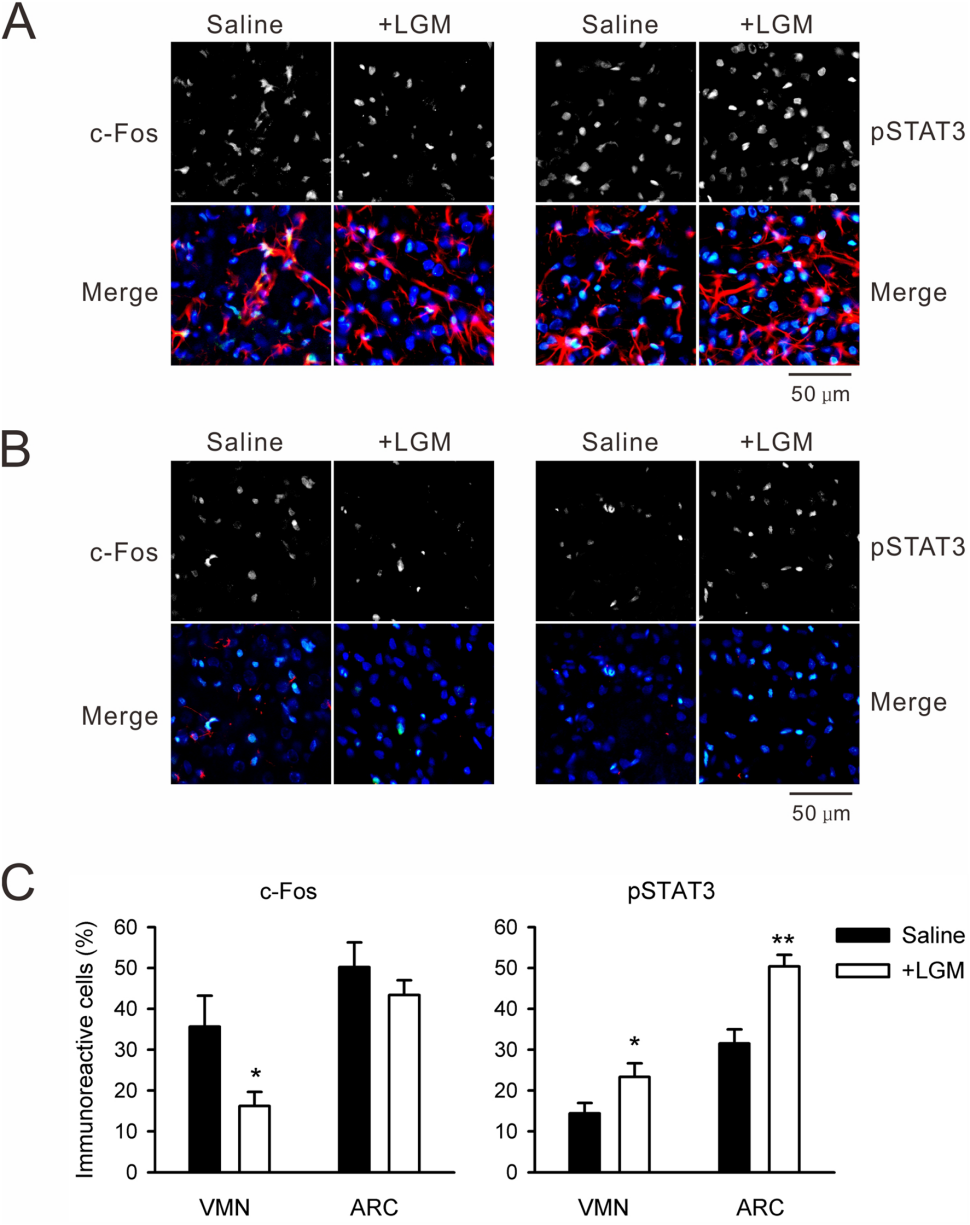
Nuclear c-Fos and pSTAT3 in the VMH. (**A**) Three hours after injection of saline or LGM (10 mg/kg, *i.p.*) as in Fig. 7B, rat brains were fixed and cryostat sections of ARC were labeled by c-Fos antibody (left-hand two panels) or pSTAT3 antibody (right-hand two panels). The image was merged with GFAP staining (red) and nuclear DAPI staining (blue). Light-blue color in the merged frames denotes c-Fos or pSTAT3 immunoreactive nuclei. (**B**) The same staining in VMN sections. The VMN contains little GFAP. (**C**) Average number of immunoreactive cells was analyzed; approximately 14–18 image frames were used to calculate the average. *p < 0.05 and **p < 0.01 compared with corresponding saline group by Student’s *t*-test. Note that c-Fos immunoreactive cells were decreased whereas pSTAT3 immunoreactive cells were increased in the VMN by LGM injection.

To investigate the cellular consequences underlying the above behavioral responses, the different groups of rats that received RF were killed 3-h after the LGM (10 mg/kg) or saline injections. Subsequently, nuclear pSTAT3-ir and c-Fos-ir were visualized in the VMH by immunohistochemistry. An astrocyte marker (glial fibrillary acidic protein, GFAP) was counterstained as a landmark of ARC because ARC is the astrocyte-rich ventral neuronal nucleus (Fig. 8A). Within the ARC, LGM injection after RF failed to modulate the number of cells displaying c-Fos-ir whereas increased number of cells displaying pSTAT3-ir (14 images in four rats, *p* < 0.01 by Student’s *t*-test; Fig. 8A,C). Within the VMN, GFAP staining was almost negligible, and LGM injection significantly decreased the number of cells displaying c-Fos-ir (18 images in four rats, *p* < 0.05 by Student’s *t*-test) whereas an increased number of cells displaying pSTAT3-ir (*P* < 0.05 by Student’s *t*-test; Fig. 8B,C).

## Discussion

CCK and leptin are both satiety-controlling signalling peptides and their synergistic roles have been reported^46–48^. A direct functional interaction between leptin and CCK signaling has been reported in peripheral nodose ganglion neurons, which are known to mediate satiety, that co-express ObRb and CCK-1 receptors^53,54^. However, a similar direct coupling has not been reported in central nervous system sites that control satiety. Here, we demonstrated such coupling could occur in the VMH as a synergistic increase in action potential firings and intracellular Ca^2+^ concentration following co-stimulation of leptin and CCK-8s. Moreover, the present study used in vitro and in vivo models to address the intracellular signalling interplay that is possibly related to satiety control and suggests a reduction in leptin-induced nuclear pSTAT3 accumulation by CCK signals.

First, the present study analysed intracellular signalling via CCK and leptin in rat C6 glioma cells. C6 cells are reported to express *ObRa* and *ObRb*^44,55^. Also, CCK-2 receptor expression has been reported in C6 cells in ligand binding studies^56^. Using real time RT-PCR and Ca^2+^ imaging assays, the present study further indicates functional CCK-1 receptor expression in a subpopulation of C6 cells. Although CCK receptor subtypes may be variable among cells, CCK-1 and CCK-2 receptors are both G_q_-coupled metabotropic receptors and thus we used C6 cells as a model to study intracellular signalling interactions via leptin and CCK receptors, irrespective of CCK receptor subtype. The results demonstrated that leptin pretreatment increased CCK-induced cytosolic Ca^2+^ mobilization. Although levels of leptin receptor expression in C6 cells may also be variable, leptin stimulation alone failed to increase cytosolic Ca^2+^ as overexpression of mouse *ObRb* had no effect. It has been reported that leptin stimulation causes cytosolic Ca^2+^ increase in cultured vagal afferent neurons^57^, hypothalamic astrocytes^44^, and porcine adrenal medullary chromaffin cells^58^. Also, in the present study, rat VMN cells in acute brain slices represented cytosolic Ca^2+^ elevation upon leptin stimulation. However, the present results in C6 cells clearly demonstrated that leptin-induced Ca^2+^ mobilization are indirect responses due to the activation of other receptor signalling molecules and/or ion channels specifically expressed in neuronal cells.

It was previously shown that ADP-induced Ca^2+^ mobilization, presumably via G_q_-coupled P2Y receptor, was enhanced by co-stimulation with leptin in megakaryoblast cells^59^. Since leptin could phosphorylate the tyrosine residue of Gqα protein by JAK2 (i.e., in AG490-dependent manner) in these cells, leptin could facilitate CCK receptor signalling similarly in C6 cells. In addition, the present study demonstrated that PI3K inhibitor, LY294002, also inhibited leptin-mediated amplification in Ca^2+^ mobilization in C6 cells. Thus, further downstream signalling might also involve the activation of phosphatidyl inositol signalling cascades.

Due to the limited permeability of peripheral CCK to the brain, the direct influence of gastrointestinal CCK on the central nervous system is still a matter of controversy. It has been suggested that CCK-mediated satiety controls are primarily mediated via the vagal afferent nerves^60,61^. However, there is also evidence that CCK stimulates satiety not only via the vagal afferent nerves but also via CCK-1 receptors beyond the blood–brain barrier, independently of the vagal nerve^15,62,63^. Indeed, hypothalamic neurons contain high levels of CCK peptides^23,24^ and CCK-1 receptors are expressed in the hypothalamic satiety-controlling axis where leptin receptors are known to be expressed^20,64^. Therefore, it is a reasonable hypothesis that intrinsic CCK signalling was enhanced by leptin in satiety-controlling hypothalamic neurons.

It was shown that leptin decreased cytosolic Ca^2+^ oscillations in primary cultures of VMN and ARC neurons^40^, whereas the present study did not identify an apparent reduction in cytosolic Ca^2+^ levels following leptin stimulation in VMN slices. In our Ca^2+^ imaging assay, all trials were performed with 0.5 µM tetrodotoxin to prevent synaptic (i.e., secondary) influences, and spontaneous Ca^2+^ oscillations were less evident. Thus, the failure to observe an inhibitory action of leptin may be due to the differential experimental conditions. Alternatively, in the present study, a monophasic Ca^2+^ increase was observed in the majority of VMN cells following leptin stimulation. Coupled with the result showing poor staining of VMN with GFAP, we suspect these responses were primarily from neurons and not from glial cells. The population analysis of Ca^2+^ imaging in the VMN indicated that there were approximately 5–12% cells that responded exclusively to leptin or CCK-8s. Therefore, the present study does not rule out the possibility that there are Ca^2+^-mobilizing machineries independent of CCK signalling cascades. However, the analysis demonstrated that more than 80% cells showing a leptin-induced Ca^2+^ increase also responded to CCK-8s, suggesting their strong association. In addition, randomly selected (by patched pipettes) VMN neurons also demonstrated bursts of action potential firing in the presence of low-concentration CCK-8s and leptin. Furthermore, cFos-ir, which is generally the result of neural excitation and Ca^2+^ responses, was significantly reduced in the VMN following LGM injection in our RF experiments. Intriguingly, the loss of the leptin-induced satiety response has been shown in VMH-lesioned rats^65^. Taken together, the positive effects of leptin on CCK-induced Ca^2+^ mobilization and action potential firing in VMN neurons could be related to the hypothalamic signalling mechanism for satiety control. Since VMN neuronal firing is also known to be sensitive to insulin and glucose^66^, it seems likely that VMN could integrate diverse satiety and/or energy controlling signals.

An important observation in C6 models, other than intracellular Ca^2+^ signalling, is the reduction in leptin-induced nuclear pSTAT3 accumulation by co-stimulation with CCK-8s. The level of pSTAT3 following co-stimulation with CCK-8s and leptin has been analyzed in primary cultures of rat nodose ganglion neurons^53,54^. In previous studies, the total abundancy of pSTAT3 but not “nuclear” pSTAT3 was quantified and a significant increase in pSTAT3 was observed by CCK-8s treatment. On the other hand, the present study demonstrated stabilisation of pSTAT3 in the cytosol of C6 cells by CCK-8s stimulation. Moreover, CCK-8s reduced the nuclear accumulation of pSTAT3 following leptin stimulation. Since this was an unexpected result in our C6 models, the present study further addressed whether such negative regulation could occur in the rat VMN. The result demonstrated *i.c.v.* co-injection of leptin and CCK-8s reduced nuclear pSTAT3 levels in the rat VMN compared with single leptin injections. Therefore, we suggest nuclear pSTAT3 as a transcriptional regulator is negatively regulated by CCK signalling, not only in model cell lines, but also in rat hypothalamic neurons.

Finally, to investigate the signalling interplay of CCK and leptin receptors in freely behaving animals, systemic injection of LGM was examined. However, pharmacological manipulation of these receptors in vivo either by agonist or antagonist injections will affect intake behaviour, which may indirectly influence their intrinsic signalling. This makes the evaluation of synergic receptor actions complex. To eliminate such secondary actions, the present study examined 1-h RF and attempted to activate intrinsic CCK and leptin signalling at steady levels before blocking of CCK-1 receptors. The results indicate: (1) LGM injection increased feeding response 3-h after injection when rats were allowed to re-feed; and (2) nuclear pSTAT3-ir was increased in the VMN and ARC by LGM injection. Thus, at least under these experimental conditions, blocking of intrinsic CCK-1 receptors signalling facilitates nuclear pSTAT3 accumulation in the VMH. This result agrees with our results in C6 models and in *i.c.v.* injection assays that indicate a reduction in nuclear pSTAT3 by CCK-8s.

Although CCK and leptin have been commonly described as satiety controlling peptides, their differential roles have also been highlighted. CCK has been shown to reduce meal frequency and ultimately offset its effects on meal size^67–69^. Unlike CCK, leptin reduces meal size but does not alter meal frequency^34,35^, suggesting a more long-term effect on body weight regulation via leptin signalling. Among the wide variety of intracellular signalling machineries linked to ObRb, it is still uncertain which pathway(s) is/are linked to what type of satiety controls. pSTAT3-ir is used as a general marker for ObRb activation because phosphorylation of STAT3 is a direct result of the JAK/STAT pathway^36,70^. Also, since pSTAT3 is a transcription factor, it is conceptually suitable for long-lasting physiological control mediated via gene expression. Here, we demonstrated reduced nuclear pSTAT3 during short-term satiety control, unlike the general leptin signalling described for satiety control. Whether such opposite outputs are used for differential regulation of short-term and long-term satiety control is an important question and further studies are needed to address this question.

## Methods

All methods were performed in accordance with the relevant guidelines and regulations. Reagent or resource information is available online as Table S1. Furthermore, the Ca^2+^ imaging protocol is described in the Supplementary Methods.

### Cell culture and gene transfection

Rat C6 glioma cells were cultured in Dulbecco’s Modified Eagle Medium/F12 (DMEM/F12) supplemented with 10% FBS, sodium bicarbonate (1.2 g/L), and 1% penicillin/streptomycin antibiotics at 37 °C in 5% CO_2_. The full-length mouse leptin receptor (*ObRb*) in pcDNA3.1 vector was kindly provided by Dr. Christian Bjørbaek (BIDMC, Harvard Medical School, Boston, MA, USA) and transfected using Lipofectamine 2000. Subsequently, the cells were cultured in medium containing 500 μg/mL G418 for cell selection.

### Animals

All procedures involving the use of animals were approved by the Institutional Animal Care and Use Committee of the University of Toyama. Male Sprague-Dawley rats were bred under a light–dark cycle (lights on 08:00–20:00 h) at a constant temperature (24 ± 1 °C) and were used for the Ca^2+^ imaging assays, immunostaining, and monitoring of feeding behavior. Water was available ad libitum. Food (regular nutrient commixture chow, Labo MR Standard) was also provided ad libitum except for the RF experiments.

### Real time RT-PCR assay

C6 cells cultured to 90% confluency in a 35 mm dish were suspended in trypsin-supplemented phosphate-buffered saline (PBS(−)), spun down at 1,000 rpm (167 × g) for 5 min, transferred to a disposable micro homogenizer tube (Nippi, Inc. BioMasher II) with 350 μl of buffer RLT (RNeasy kit), and homogenized at 2,500 rpm for 30 s. Following the addition of 350 μl of 70% ethanol, samples were stored at − 80 °C. Total RNA (4 μg per sample) was extracted from each tissue homogenate. Reverse transcription, including DNase treatment, was performed using a QuantiTect reverse transcription kit with standard procedures. PCR primers were designed according to Hsuchou et al.^55^ for ObRa and ObRb receptors and Ko et al.^71^ for CCK-1 and CCK-2 receptors as follows: rat *ObRa* forward primer, TGAAGTATCTCATGACCACTACAGATGA; rat *ObRa* reverse primer, GTTTGCTTCCTTCCTTCAAAATGT; rat *ObRb* forward primer, GCATGCAGAATCAGTGATATTTGG; rat *ObRb* reverse primer, CAAGCTGTATCGACACTGATTTCTTC; rat CCK-1 receptor forward primer, CAGCAGGCCGGTGATAAGA; rat CCK-1 receptor reverse primer, GGTGGACATGAGAAGGTGT; rat CCK-2 receptor forward primer, CGCCATATGCCGACCACTG; rat CCK-2 receptor reverse primer, CCACACCCGGGATGAAGAAC; *GAPDH* forward primer, GGCACAGTCAAGGCTGAGAATG; *GAPDH* reverse primer, ATGGTGGTGAAGACGCCAGTA. Real time PCR was performed using the Rotor Gene 3000A system with a 72-well rotor, as described previously^72^.

### Electrophysiology

The VMH slices (300-μm thickness) were prepared following deep pentobarbital anaesthesia administration to postnatal day (PD) 12–16 rats using a vibrating blade microtome in ice-cold high-Mg^2+^ artificial cerebrospinal fluid (ACSF) containing 138.6 mM NaCl, 3.35 mM KCl, 21 mM NaHCO_3_, 0.6 mM NaH_2_PO_4_, 9.9 mM d-glucose, 0.5 mM CaCl_2_, and 4 mM MgCl_2_ bubbled with 95% O_2_/5% CO_2_. The slices were incubated at room temperature for 1 h in regular ACSF containing 2.5 mM CaCl_2_, and 1.0 mM MgCl_2_ bubbled with 95% O_2_/5% CO_2_. Subsequently, the slices were transferred to an upright microscope stage (Olympus BX50WI). The recording chamber was perfused with oxygenated normal ACSF at 3 ml/min at 34 °C. VMN neurons were visualized through an infrared CCD camera (Hamamatsu Photonics C2741-79). Neurons were recorded in the whole-cell current-clamp mode using a patch-clamp amplifier (Axon Instruments Axopatch 200B). The recordings were performed using patch pipettes with an access resistance of 4–6 MΩ and internal solution containing 135 mM potassium gluconate, 5 mM KCl, 10 mM HEPES, 0.1 mM CaCl_2_, 1.0 mM EGTA, 10 mM phosphocreatine, 2 mM Mg-ATP, and 0.2 mM Na-GTP (pH adjusted to 7.3 with KOH). The output of the amplifier was digitised using an A/D converter board (Axon Instruments Digidata 1200) with a sampling rate of 10 kHz and recorded on a hard disk by data acquisition software (Axon Instruments pCLAMP 8). Membrane potentials were low-pass-filtered at 2 kHz. Resting membrane potentials less than − 60 mV were set to approximately − 60 mV by current injections. Stimulants were applied by the gravity drop perfusion system. Further details of electrophysiological recordings were described previously^73^.

### I.c.v. administration of leptin and CCK

A guide cannula (cut length 5 mm, no. C315G; Plastics One Inc.) was stereotaxically implanted to the right lateral ventricle of each rat, with the coordinates of 0.8 mm posterior to the bregma, 1.4 mm lateral to the midline, and 3.3 mm ventral from the skull surface. The canula was secured on the skull surface with two stainless steel screws and dental acrylic resin. The rats were then allowed to recover in their home cage for at least 1 week. Leptin (4 µg) and CCK (2 µg) were dissolved in 4 µl ACSF and administered through an internal cannula (cut length 5.5 mm, no. C315I; Plastics One Inc. C315I). To minimize the effect of endogenous signals, the rats were deprived of food for 10 h (08:00–18:00) before *i.c.v.* administration. Drugs were infused at a flow rate of 1 µl/min over 4 min using a motorized syringe pump (Carnegie Medicine model CMA/102), followed by another 4 min of rest for diffusion. One hour after *i.c.v.* administration, the rat brain was removed for immunohistochemical analysis.

### Immunofluorescent confocal imaging

To examine the effects of leptin and CCK-8s on STAT3 phosphorylation levels, C6-ObRb at 60–70% confluency on 35 mm glass-bottom dishes were treated with leptin (100 nM) or CCK-8s (10 nM) 30 min prior to fixation. Cells were treatment with AG490 (1 μM) or LY294002 (50 μM) 15 min prior to the leptin stimulation until the time of fixation. These cells were fixed in 4% phosphate-buffered paraformaldehyde for 15 min and washed three times with PBS(−). The fixed samples were then incubated for 2 h at room temperature in 10% donkey serum dissolved in 0.1% Triton-X PBS(−). Next, samples were incubated with 1:100 affinity-purified rabbit phospho-STAT3 (pTyr^705^) dissolved in 5% donkey serum PBS(−) for 24 h at 4 °C. After three 20-min PBS(−) rinses, samples were incubated in 1:400 Cy3-conjugated donkey anti-rabbit IgG for 2 h at room temperature. Finally, samples were rinsed with PBS(−) (four 15-min rinses on an orbital shaker) and mounted using Vectashield containing 4′,6-diamidino-2-phenylindole (DAPI).

For immunohistochemical analysis of rat hypothalamic slices, animals were deeply anesthetized with an *i.p.* injection of sodium pentobarbital (50 mg/kg) and transcardially perfused with PBS(−) for 5 min followed by ice-cold 4% (w/v) paraformaldehyde in PBS(−) for 15 min. The brain was removed and further fixed in the same fixative (4 °C, overnight). Then, the olfactory bulbs and/or cerebellum were removed from the brain in ice-cold PBS(−). Pre-trimmed brain tissues were immersed in 30% (w/v) sucrose and stored overnight at 4 °C. The fixed and cryoprotected brain tissues were embedded with OCT compound. Frozen sections of 30 µm thickness were cut using a cryostat microtome and washed three times with PBS(−) in 24-well plates, after which they were mounted on glass slides. The cryostat brain sections were then stained as above for anti-phospho-STAT3 or 1:5,000 anti-c-Fos (AB-5) rabbit pAb with 1:200 Alexa488-conjugated donkey anti-rabbit IgG. Finally, the samples were incubated with 1:200 Cy3-conjugated mouse anti-GFAP, rinsed thoroughly with PBS(−) and mounted using Vectashield containing DAPI.

Images were acquired using a confocal laser-scanning microscope (Olympus FV1000 or Nikon A1R MP plus) with a laser diode (405 nm), argon laser (488 nm), and helium neon laser (534 nm). The nuclear immunofluorescent intensity (8-bit depth) was analyzed using Photoshop CS 6 software.

### RF and counting of food accesses

Male rats aged 2–3 months were individually housed in originally designed cylindrical chambers (30 cm ϕ, H30 cm). Food was given ad libitum from slits of stainless pellet server placed 22 cm above the floor. Before counting of food accesses, animals were fed according to the 1-h RF paradigm shown in Fig. 7A. During the RF, rats were fed with fatty food paste composed of 50% nutrient commixture chow powder, 20% soybean oil, and 30% water on a glass dish. Following removal of the dish, sterilized saline (1 ml/kg body weight) were *i.p.* injected. Leftover food paste was weighed after RF to calculate daily intake. Rats were acclimatized to this environment for 4 days. On day 5 of RF, saline or LGM (2 or 10 mg/kg body weight) dissolved in saline was injected. Three hours after the drug injection, rats were re-fed with regular chow in a previously setup pellet server. A touch sensing probe (Elekit PS-306) was connected to the pellet server. Because the bottom of the pellet server was placed 22 cm above the floor, purposeful food access behavior, but not general locomotor activities, was counted using this system. The ON/OFF signals from the sensor were fed into a laptop computer through a photo-coupler isolated digital I/O card (Contec Inc. PIO-16/16L) and automatically counted at 3-min intervals by software written by M.I.

### Statistical analysis

Data are presented as means with standard error. Two-tailed Student’s *t*-test was used for the pair wise comparisons. One-way analysis of variance (ANOVA) followed by Duncan’s multiple range tests were used for the statistical comparison across multiple means of single factor. Two-way ANOVA was used to compare multiple concentration response curves. A four-parameter Hill function was used to estimate the concentration response curve. A 95% confidence level was considered to indicate statistical significance.

## Supplementary information

Supplementary Information
