# Supplementary material for: Intracellular interplay between cholecystokinin and leptin signalling for satiety control in rats
Source: Sci Rep. 2020 Jul 20;10:12000. doi: 10.1038/s41598-020-69035-6 (PMC7371863; doi:10.1038/s41598-020-69035-6)
Supplement: Supplementary file 1 — Supplementary Information [file 41598_2020_69035_MOESM1_ESM.docx]

**SupplementaRY Information**

for

Intracellular interplay between cholecystokinin and leptin signalling for satiety control in rats

Hayato Koizumi^1,2^, Shahid Mohammad^1,3^, Tomoya Ozaki^1,4^, Kiyokazu Muto^2^, Nanami Matsuba^2^, Juhyon Kim^1,2^, Weihong Pan^5#^, Eri Morioka^2^, Takatoshi Mochizuki^2^ & Masayuki Ikeda^1,2^*

*^1^Graduate School of Innovative Life Science, University of Toyama, 3190 Gofuku, Toyama 930-8555, Japan*

*^2^Graduate School of Science and Engineering, University of Toyama, 3190 Gofuku, Toyama 930-8555, Japan*

*^3^Center for Neuroscience Research, Children's National Medical Center, Washington, DC 20010, USA*

*^4^Graduate School of Medicine, Nagoya University, 65 Tsurumaicho, Showa-ku 466-8550, Japan*

*^5^Blood-Brain Barrier Group, Pennington Biomedical Research Center, 6400 Perkins Road, Baton Rouge, LA 70808, USA*

*^#^Current address: Biopotentials Consulting, Sedona, AZ 86351, USA*

***Correspondence:** msikeda@sci.u-toyama.ac.jp

**This PDF file includes:**

Figure S1

Table S1

References

Supplementary methods

**
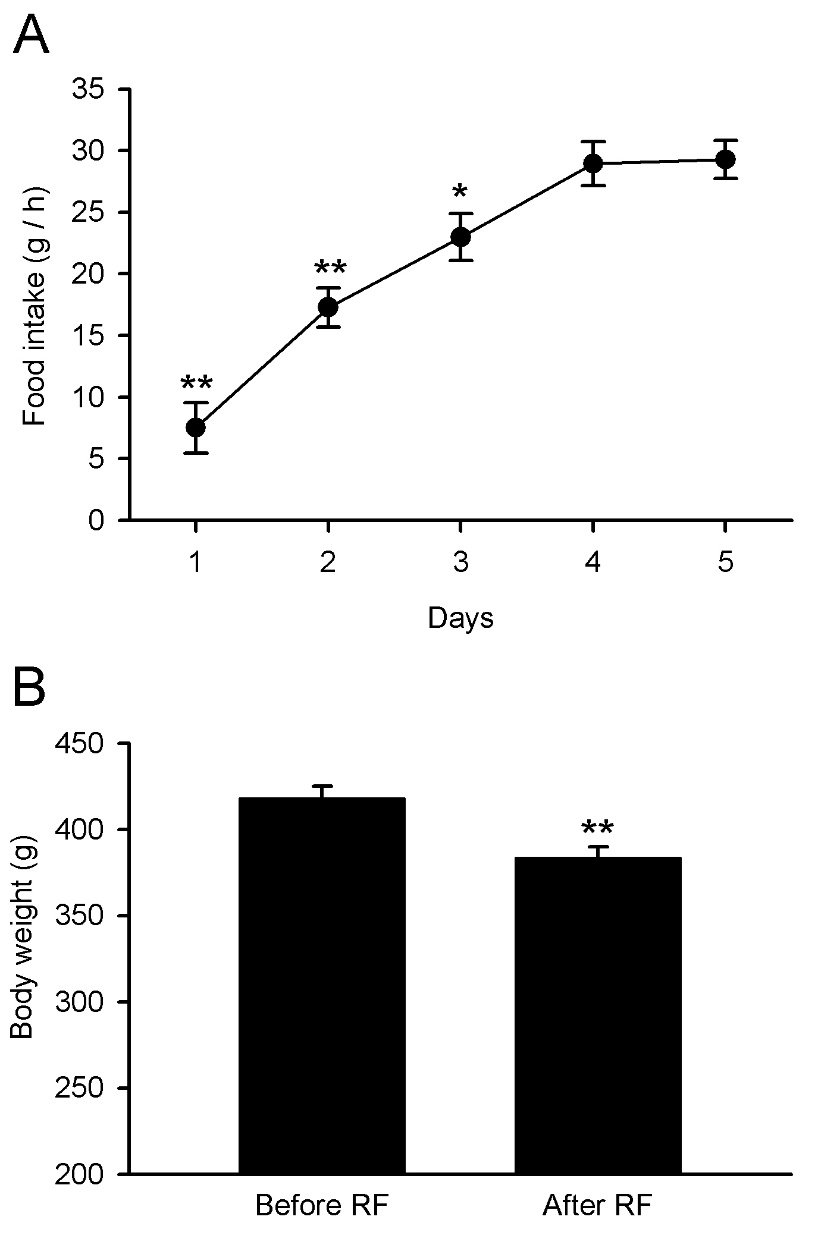
**

**Figure S1 A.** Daily food intake volume during the restricted feeding (RF) period. Intake volume during the 1-h RF in Figure 7 is shown. Rats acclimatized to the RF and stabilised their meal size during the 5-day period. The intake volumes on day 4 and 5 were not significantly different but were larger than the volumes during the first 3 days (***P*<0.01 and **P*<0.05 by Duncan’s multiple range test following one-way ANOVA). **B.** Body weight of rats before and after the 5-day RF period. After the RF, body weight was reduced by approximately 8%. ***P*<0.01 by two-tailed paired *t*-test.

**Table S1. Reagent or resource information including their source and identifiers.**

| REAGENT or RESOURCE | SOURCE | IDENTIFIER |
| --- | --- | --- |
| Antibodies | | |
| Rabbit phospho-STAT3 (pTyr^705^) | Sigma-Aldrich  (St. Louis, MO, USA) | Cat#S2690  RRID:AB_262055 |
| Donkey anti-rabbit IgG - Cy3 | Jackson ImmunoResearch (West Grove, PA, USA) | Cat#711-165-152;  RRID:AB_2307443 |
| Donkey anti-rabbit IgG - Alexa Fluor 488 | Jackson ImmunoResearch | Cat#711-545-152;  RRID:AB_2313584 |
| Mouse anti-glial fibrillary acidic protein - Cy3 | Sigma-Aldrich | Cat#G3893;  RRID:AB_ 477010 |
| Rabbit anti-c-Fos (Ab-5) | Calbiochem (San Diego, CA, USA) | Cat#PC38T;  RRID:AB_ 2106755 |
| Chemicals, Peptides, and Recombinant Proteins | | |
| DMEM/F12 | Invitrogen (Carlsbad, CA, USA) | Cat#12400-024 |
| FBS | Wako Pure Chemical Industries  (Osaka, Japan) | Cat#535-94155 |
| Sodium bicarbonate | Sigma-Aldrich | Cat#S5761 |
| Penicillin-streptomycin | Invitrogen | Cat#15140-122 |
| G418 disulfate salt | Sigma-Aldrich | Cat#A1720 |
| 2.5% Trypsin | Invitrogen | Cat#15090-046 |
| Ethanol | Wako Pure Chemical Industries | Cat#057-00456 |
| NaCl | Wako Pure Chemical Industries | Cat#195-01663 |
| KCl | Wako Pure Chemical Industries | Cat#163-03545 |
| CaCl_2_ | Wako Pure Chemical Industries | Cat#031-00435 |
| MgCl_2_ | Wako Pure Chemical Industries | Cat#132-00175 |
| D-glucose | Wako Pure Chemical Industries | Cat#041-00595 |
| HEPES | Wako Pure Chemical Industries | Cat#346-01373 |
| NaOH | Wako Pure Chemical Industries | Cat#192-15985 |
| Fura 2-AM | Dojindo Laboratories (Kumamoto, Japan) | Cat#F015 |
| NaH_2_PO_4_ | Wako Pure Chemical Industries | Cat#192-02815 |
| Pluronic F-127 | Invitrogen | Cat#P6867 |
| Tetrodotoxin | Wako Pure Chemical Industries | Cat#207-15901 |
| Leptin | PeproTech (Rocky Hill, NJ, USA) | Cat#450-31 |
| CCK-4 | Peptide Institute, Inc. (Osaka, Japan) | Cat#4083-V |
| CCK-8s | Sigma-Aldrich | Cat#C2175 |
| AG490 | Calbiochem | Cat#658401 |
| LY294002 | Calbiochem | Cat#440202 |
| LY225910 | R&D Systems (Minneapolis, MN, USA) | Cat#1018/10 |
| Lorglumide | LKT Laboratories Inc. (St. Paul, MN, USA) | Cat#L5769 |
| ATP disodium salt hydrate | Sigma-Aldrich | Cat#S26209 |
| Potassium gluconate | Sigma-Aldrich | Cat#S2054 |
| EGTA | Sigma-Aldrich | Cat#E4378 |
| Phosphocreatine disodium salt hydrate | Sigma-Aldrich | Cat#P7936 |
| Mg-ATP | Sigma-Aldrich | Cat#A9187 |
| Na-GTP | Sigma-Aldrich | Cat#G8877 |
| 1 mol/l KOH | Wako Pure Chemical Industries | Cat#169-03885 |
| Paraformaldehyde | Wako Pure Chemical Industries | Cat#162-16065 |
| Normal donkey serum | Jackson ImmunoResearch | Cat#017-000-121 |
| TritonX-100 | Sigma-Aldrich | Cat#T9284 |
| Vectashield | Vector Laboratories (Burlingame, CA, USA) | Cat#H-1200 |
| Pentobarbital Sodium Salt | Tokyo Chemical Industry (Tokyo, Japan) | Cat#P0776 |
| OCT compound | Sakura Finetek (Tokyo, Japan) | Cat#45833 |
| Soybean oil | Wako Pure Chemical Industries | Cat#190-03776 |
| Otsuka normal saline | Otsuka Pharmaceutical (Tokyo, Japan) | Cat#7131400A2013 |
| Experimental Models: Cell Lines | | |
| Rat: C6 glioma cells |  |  |
| Experimental Models: Organisms/Strains | | |
| Rat: Crl:CD(SD) | Charles River Laboratories Japan (Yokohama, Japan) | Cat#001A11 |
| Oligonucleotides and Recombinant DNA | | |
| Full length mouse leptin receptor | Bjørbaek et al., 1997 | N/A |
| pcDNA3.1(-) vector | Invitrogen | Cat#V795-20 |
| Primer: rat ObRa Forward: TGAAGTATCTCATGACCACTACAGATGA | Hsuchou et al., 2009 | N/A |
| Primer: rat ObRa Reverse: GTTTGCTTCCTTCCTTCAAAATGT | Hsuchou et al., 2009 | N/A |
| Primer: rat ObRb Forward: GCATGCAGAATCAGTGATATTTGG | Hsuchou et al., 2009 | N/A |
| Primer: rat ObRb Reverse: CAAGCTGTATCGACACTGATTTCTTC | Hsuchou et al., 2009 | N/A |
| Primer: rat CCK-1 receptor Forward: CAGCAGGCCGGTGATAAGA | Ko et al, 2006 | N/A |
| Primer: rat CCK-1 receptor Reverse: GGTGGACATGAGAAGGTGT | Ko et al, 2006 | N/A |
| Primer: rat CCK-2 receptor Forward: CGCCATATGCCGACCACTG | Ko et al, 2006 | N/A |
| Primer: rat CCK-2 receptor Reverse: CCACACCCGGGATGAAGAAC | Ko et al, 2006 | N/A |
| Primer: rat GAPDH Forward: GGCACAGTCAAGGCTGAGAATG |  | NM_017008.4 |
| Primer: rat GAPDH Reverse: ATGGTGGTGAAGACGCCAGTA |  | NM_017008.4 |
| Other |  |  |
| Labo MR Standard | Nosan Corporation (Yokohama, Japan) |  |
| Lipofectamine-2000 | Invitrogen | Cat#11668-019 |
| RNeasy Mini Kit | Qiagen (Chatsworth, CA, USA) | Cat#74106 |
| QuantiTect Reverse Transcription Kit | Qiagen | Cat#205311 |
| Rotor-Gene SYBR Green PCR Kit | Qiagen | Cat#204074 |
| Software and Algorithms | | |
| Rotor-Gene Ver. 6 | Corbett Research (Mortlake, NSW, Australia) | N/A |
| MetaFluor ver. 6.2 | Japan Molecular Devices  (Tokyo, Japan) | N/A |
| Origin 5.0 | OriginLab (Northampton, MA, USA) | N/A |
| pCLAMP 8 | Axon Instruments (Union City, CA, USA) | N/A |
| Photoshop CS 6 | Adobe Systems (San Jose, CA, USA) | N/A |
| Equipments | | |
| BioMasher II | Nippi, Inc. (Tokyo, Japan) | Product#320 102 |
| BX50WI | Olympus Corporation (Tokyo, Japan) | N/A |
| C2741-79 | Hamamatsu Photonics (Hamamatsu, Japan) | N/A |
| C315G/SPC | Plastics One, Inc. (Roanoke, VA, USA) | ICN:8IC315GSPCXC |
| C315I/SPC | Plastics One, Inc. | ICN:8IC315ISPCXC |
| CMA/102 | Carnegie Medicine (Stockholm, Sweden) | N/A |
| FLUOVIEW FV1000 | Olympus Corporation | N/A |
| A1R MP plus | Nikon Corporation (Tokyo, Japan) | N/A |
| PS-306 | Elekit Japan Co., Ltd. (Fukuoka, Japan) | N/A |
| PIO-16/16L(PM) | Contec, Inc. (Tokyo, Japan) | N/A |

**References**

1. Bjørbaek, C., Uotani, S., da Silva, B., Flier, J. S. Divergent Signaling Capacities of the Long and Short Isoforms of the Leptin Receptor. *J. Biol. Chem.* **272**, 32686-95 (1997).
2. Hsuchou, H., Pan, W., Barnes, M. J. & Kastin, A. J. Leptin receptor mRNA in rat brain astrocytes. *Peptides* **30**, 2275-2280. (2009).
3. Ko, E. S., Kim, S. K., Kim, J. T., Lee, G., Han, J. B., Rho, S. W., Hong, M. C., Bae, H. & Min, B. I. The difference in mRNA expressions of hypothalamic CCK and CCK-A and -B receptors between responder and non-responder rats to high frequency electroacupuncture analgesia. *Peptides* **27**, 1841-1845 (2006).

**Supplementary methods**

Ca^2+^ imaging

C6 cells plated on 35 mm plastic dishes were gently rinsed with buffered salt solution (BSS) consisting of 130 mM NaCl, 5.4 mM KCl, 2 mM CaCl_2_, 1 mM MgCl_2_, 5.5 mM D-glucose, and 10 mM HEPES/NaOH (pH 7.4), and incubated in BSS supplemented with 6 µM Fura-2 AM (Dojindo Laboratories, Kumamoto, Japan) for 30 min in a CO_2_ incubator. Prior to photometry, the cells were rinsed again with BSS, incubated at 37°C for 15 min, and then continuously perfused with BSS at 34°C at a flow rate of 3 mL/min. Fluorescence images were obtained using an upright microscope (Axioplan2; Carl Zeiss, Thornwood, NY, USA) with a water-immersion objective (Achroplan×20 NA0.5w; Carl Zeiss). The wavelength of the excitation UV light (340 nm or 380 nm pulse; 250 msec) was switched using a filter wheel (Lambda 10-2; Sutter Instruments, Novato, CA, USA). The UV light was generated by a full spectrum 300W Xenon bulb (Lambda LS; Sutter Instruments), conducted to the microscope through a liquid light guide. The pair of fluorescent images was processed using a band-pass filter (485–515 nm) and exposed to a multiple format cooled CCD camera (CoolSnap-fx; Photometrics, Tucson, AZ, USA) at 6-sec intervals. The filter wheel and the CCD camera were controlled using digital imaging software (MetaFluor ver. 6.2; Japan Molecular Devices, Tokyo, Japan). The background fluorescence was also subtracted using the software.

For Ca^2+^ imaging using hypothalamic slices, coronal hypothalamic slices (300 µm) were prepared following deep pentobarbital anesthesia on postnatal day (PD) 10–12 rats using a vibrating blade microtome in ice-cold high-Mg^2+^ artificial cerebrospinal fluid (ACSF) containing 138.6 mM NaCl, 3.35 mM KCl, 21 mM NaHCO_3_, 0.6 mM NaH_2_PO_4_, 9.9 mM D-glucose, 0.5 mM CaCl_2_, and 4 mM MgCl_2_, and bubbled with 95% O_2_/5% CO_2_. The slices were incubated at room temperature for 1–4 hours in regular ACSF containing 138.6 mM NaCl, 3.35 mM KCl, 21 mM NaHCO_3_, 0.6 mM NaH_2_PO_4_, 9.9 mM D-glucose, 2.5 mM CaCl_2_, and 1.0 mM MgCl_2_ bubbled with 95% O_2_/5% CO_2_. For Ca^2+^ imaging, the slices were placed in a 0.40 µm filter cup (Millicell-CM, Millipore, Berford, MA, USA) and immersed for 45–60 min in regular ACSF containing 10 µM Fura-2 AM (Dojindo Laboratories) and 0.01% w/v Pluronic F-127 (Invitrogen). During the entire staining procedure, the staining solution was gently bubbled with 95% O_2_/5% CO_2_ through a stainless-steel pipe located outside the cup filter. After washout of the staining solution with regular ACSF and incubating for an additional 30 min, the slice was gently removed from the holding membrane filter and placed in a glass-bottomed microscope stage chamber (0.5 mL) for optical measurement of cytosolic Ca^2+^ concentration. During recording, slices were perfused with ACSF containing tetrodotoxin (0.5 µM, Wako Pure Chemical Industries) at 34°C at a flow rate of 2.5 mL/min.

During image acquisition, leptin (PeproTech, Rocky Hill, NJ, USA), CCK-4 (Peptide Institute, Inc., Osaka, Japan), CCK-8s (Sigma-Aldrich, St. Louis, MO, USA), AG490 (Calbiochem, San Diego, CA), LY294002 (Calbiochem), LY225910 (R&D Systems, Minneapolis, MN, USA), LGM (LKT Laboratories Inc., St. Paul, MN, USA), and ATP (Sigma-Aldrich) were applied by switching the perfusate. The magnitude of Ca^2+^ responses was determined by peak area analysis using Origin 5.0 software (OriginLab, Northampton, MA, USA).
